# Forest age estimation in northern Arkhangelsk region based on machine learning pipeline on Sentinel-2 and auxiliary data

**DOI:** 10.1038/s41598-023-49207-w

**Published:** 2023-12-13

**Authors:** Alina Smolina, Svetlana Illarionova, Dmitrii Shadrin, Alexander Kedrov, Evgeny Burnaev

**Affiliations:** 1 Skolkovo Institute of Science and Technology, Applied AI Center, Moscow, Russia 121205; 2 Space Technologies and Services Center, Ltd, Perm, Russia 614038; 3 Autonomous Non-Profit Organization Artificial Intelligence Research Institute (AIRI), Moscow, Russia 105064

**Keywords:** Ecology, Environmental sciences

## Abstract

Tree age is one of the key characteristics of a forest, along with tree species and height. It affects management decisions of forest owners and allows researchers to analyze environmental characteristics in support of sustainable development. Although forest age is of primary significance, it can be unknown for remote areas and large territories. Currently, remote sensing (RS) data supports rapid information gathering for wide territories. To automate RS data processing and estimate forest characteristics, machine learning (ML) approaches are applied. Although there are different data sources that can be used as features in ML models, there is no unified strategy on how to prepare a dataset and define a training task to estimate forest age. Therefore, in this work, we aim to conduct a comprehensive study on forest age estimation using remote sensing observations of the Sentinel-2 satellite and two ML-based approaches for forestry inventory data, namely stand-based and pixel-based. We chose the CatBoost algorithm to assess these two approaches. To establish the robustness of the pipeline, an in-depth analysis is conducted, embracing diverse scenarios incorporating dominant species information, tree height, Digital Elevation Model (DEM), and vegetation indices. We performed experiments on forests in the northern Arkhangelsk region and obtained the best Mean Absolute Error (MAE) result of 7 years in the case of the stand-based approach and 6 years in the case of the pixel-based approach. These results are achieved for all available input data such as spectral satellites bands, vegetation indices, and auxiliary forest characteristics (dominant species and height). However, when only spectral bands are used, the MAE metric is the same both for per-pixel and per-stand approaches and equals 11 years. It was also shown that, despite high correlation between forest age and height, only height can not be used for accurate age estimation: the MAE increases to 18 and 26 years for per-pixel and per-stand approaches, respectively. The conducted study might be useful for further investigation of forest ecosystems through remote sensing observations.

## Introduction

Forest age is an important proxy for forest management and conservation^1^. It is used for timing of management activities, harvesting and estimation of resource stocks (such as carbon or timber). Forest age is vital in ecological studies as it influence on soil water behaviour and hydraulic conductivity^2^ and helps to estimate wildfire risk^3^. Together with other forest parameters, forest age is usually measured by means of field studies; however, these methods are labour-intensive and not economically feasible on a large scale^4^. Advanced sensing techniques allow one to collect Earth observations with required spatial–temporal properties^5^. Therefore, contemporary approaches in environmental studies are focused on remote sensing data implementation in forest properties estimation tasks^6^.

A common pipeline to estimate forest age usually follows this sequence: an intermediate variable such as height, stock volume, crown projection area, or another is estimated from remote sensing data and then converted to age. For instance, forest age is highly correlated with forest height^7^. The development of biological systems over time is associated with their growth and is strongly dependent on plants, particularly trees. Therefore, during growth and maturation a tree age and height can change according to the same dynamics, and a number of studies used this relationship to estimate the age of the tree^8^. Mostly, linear dependency between age and height is used, as in Ref.^9–11^. However, several studies have analyzed nonlinear age–height relationships, such as^12–14^. For such approaches, the forest height data can be acquired from Light Detection and Ranging (LiDAR) measurements, filed-based measurements, or can be artificially derived from multispectral satellite data. In Ref.^12^, they aimed to generate a national map of forest age in China with a spatial resolution of 1 km using remotely sensed data and age-height relationships derived from field observations. Results were reported on regional ($R^2 = 0.87$, $\text {RMSE}=4$ years) and provincial levels ($R^2 = 0.53$, $\text {RMSE}=12$ years). The study^14^ is an enhancement of the approach introduced in Ref.^12^ with additional using of climate data. Climate data (annual precipitation and mean annual temperature) was used to refine the age–height relationships derived from field observations. The age was then calculated using the age–height relationships obtained with the LiDAR derived height. Another proposed scheme^9^ produces a continuous forest age map at a regional scale. Field data were used to establish the parameters of a linear equation between age and height. Then, the discrete point dataset of average tree height from ICESat/GLAS data (or equivalent LiDAR derived height) was extended to a regional scale continuous height map using MODIS BRDF data. This map is used for the final forest age determination. In Ref.^15^, height-to-age conversions were determined independently for each tree species and site index with reported RMSE of 11.5 years. However, without the corresponding maps, this approach cannot be applied to new areas.

Apart from age-height relationship modelling, there are studies that instead of height use other forest characteristics as main predictor variable. In Ref.^16^, authors first estimated the growing stock volume (using the kNN method) on optical remote sensing data and field data for those pixels without field data. The age was then obtained as a function of the growing stock, the parameters of this relationship were obtained from the inventory data. In Ref.^11^, for oil palm age estimation, authors proposed to use a crown projection area which was estimated from high resolution multispectral data. Although such auxiliary forest variables can provide valuable information for age estimation, they are typically limited in practice.

The main limitation of such approaches is that the growth rate of each tree type is assumed a fixed value, regardless environmental conditions^17^ and correlation functions should be estimated and refined for new ares. A less commonly discussed approach is based on direct forest age estimation through satellite data. For instance, in Ref.^18^, Landsat TM and SPOT HRV imagery were implemented to estimate forest age both on stand and pixel levels. In Ref.^19^, the authors investigated various combinations of multiple open access data sources to predict forest characteristics including forest age. The achieved RMSE for forest age estimation in this study equals to 17%. Sentinel-1 and Sentinel-2 observations have shown to be a promising data source for forest age mapping into 6 age classes with an overall accuracy of 70%^20^. Although satellite data is widely used for estimating other forest characteristics such as tree species and height, there has been less research on predicting forest age through multispectral freely available images with medium spatial resolution. Instead, forest age prediction has mostly relied on auxiliary taxation measurements that might be limited for certain areas. Therefore, in this study, we set an objective to investigate capabilities of freely available Sentinel-2 images in a several algorithmic setups.

To develop an ML-based pipeline for forest age estimation, one should specify the remote sensing data, an ML algorithm, and strategies to train and verify this algorithm (such as defining a training sample in a dataset and splitting a datasets into train, validation, and test sets). Even for one data source there is not a single unified approach to solve the task. The remote sensing domain specificity should also be taken into account, namely spatial distribution of studied objects. Therefore, a comprehensive study of different feature spaces and training strategies is required. In this work, we focus on middle-resolution multispectral Sentinel-2 data, which is a common choice for local and global studies of forest cover due to its wide spectral features, open access, and relatively detailed spatial resolution. Our goal is to investigate the usage of Sentinel-2 data with the auxiliary data, such as DEM measurement, in two ML pipelines for pixel-based and stand-based age estimation. We also consider two approaches to spatially distributed testing and training splits. To gain a deeper understanding of the significance of different features and ML strategies, we consider four tree species in the northern region.

**Figure 1:**
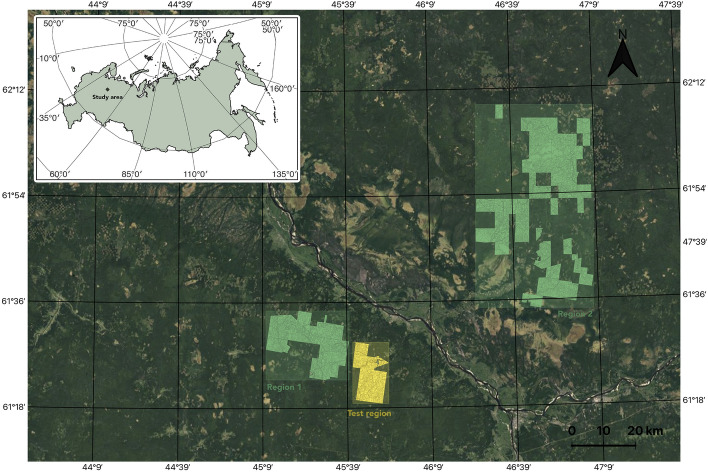
Study area and regions with available forest inventory data. Yellow region (Test region) corresponds to $\text {Test}_{\text {forestry}}$. Green regions 1 and 2 is randomly split into training, validation, and $\text {Test}_{\text {random}}$ sets. The map was generated with the QGIS v.3.14 software (https://qgis.org/en/site/) and RGB satellite composite from Google Maps layers available in QGIS.

## Methods

### Forest inventory data analysis

The study area is located in the north of the European part of Russia, in the Arkhangelsk region. The relative location is shown in Fig. 1. The region of interest has coordinates between $45^\circ \, 9^{\prime }$ E and $47^\circ \, 9^{\prime }$ E longitude and between $61^\circ \, 18^{\prime }$ N and $62^\circ \,12^{\prime }$ N latitude.

**Figure 2:**
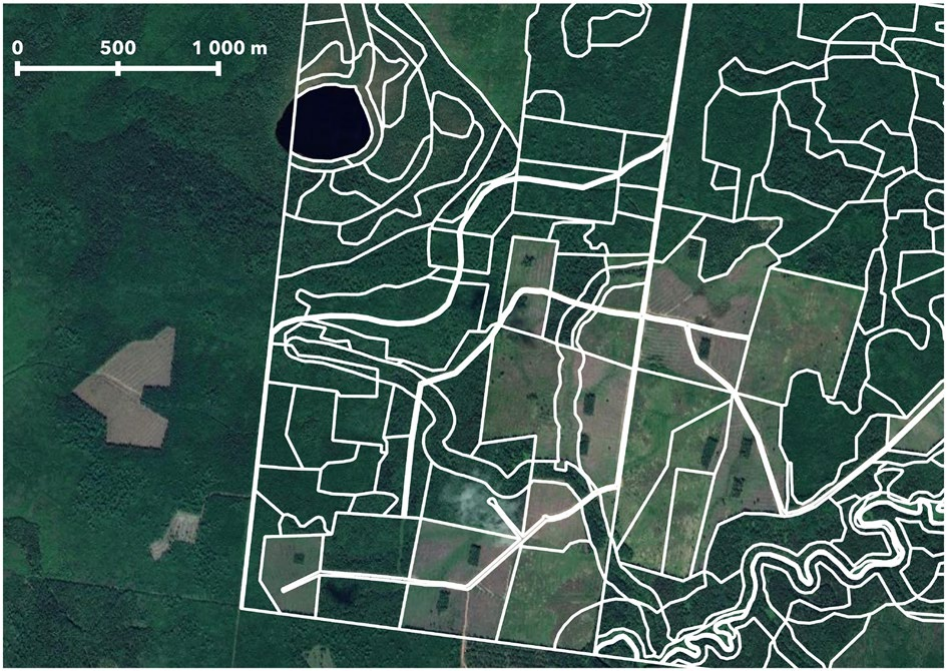
Example of used forest inventory data with boundaries of individual forest stands. The map was generated with the QGIS v.3.14 software (https://qgis.org/en/site/) and RGB satellite composite from Google Maps layers available in QGIS.

The forestry inventory data was collected in 2018 for several forestries in Arkhangelsk region, covering a total area of 126–641 ha. It is presented as vector georeferenced data and includes boundaries for 12,617 forest stands. Average forest stand size is 10 ha. The term *forest stand* refers to a specific area or section of a forest that is relatively uniform in terms of its composition and structure^21^. Each individual stand is supplied with information about main forest characteristics. Figure 2 depicts several stands in inventory data.

In this study, we consider the following forest characteristics:

- *Forest stand species structure.* The composition formula is ascertained by calculating the percentage of each tree species presented in the stand. This composition is presented in percentage points (composition coefficients), where each point equates to a ratio of 10% of total stand stock. We use forest composite formula to define dominant species (species with the content more than 50% within a stand).
- *Mean stand age (years).* The average age of *model trees* belonging to the given stand (for a fixed type). A model tree is an average tree in its parameters, in our case such a parameter was the diameter at breast height (DBH). It should be noted, that trees with DBH $\ge 1$ cm are recorded in the forest inventory data.
- *Mean stand height (m).* The average height of the dominant part of the stand (weighted average height between the species involved in the composition of the stand, based on composition coefficients).

Seven species types are presented in stand structure data: spruce, birch, pine, fir, aspen, willow and alder (the scientific names for the species are outlined in Table 1), however, not all of these species are present in large enough numbers. To account for only the most prevalent tree species, we consider the dominant forest species, which is a species that occurs in the formula at a frequency greater than 50%. If there is no dominant species or if two species occur equally, the stand is considered mixed.

**Table 1:** The scientific names with authorities for the species cited in the study.

Name in the study	Vernacular name	International scientific name
Spruce	Norway spruce	*Picea abies* (L.) H. Karst.
Spruce	Finnish spruce	*Picea fennica* (Regel) Kom.
Birch	European white birch	*Betula pendula* Roth
Pine	Scots pine	*Pinus sylvestris* L.
Fir	Siberian fir	*Abies sibirica* Ledeb.
Aspen	European aspen	*Populus tremula* L.
Alder	Gray alder	*Alnus incana* (L.) Moench
Willow	Goat willow	*Salix caprea* L.
Willow	Bay willow	*Salix pentandra* L.
Willow	Almond willow	*Salix triandra* L.

The stands with a dominant spruce species are prevailing (8–532; 68%). There are also (1–745; 14%) stands of birch, (541; 4%) stands of pine and (370; 3%) stands of aspen, other stands are considered mixed (1–429; 11%).

**Figure 3:**
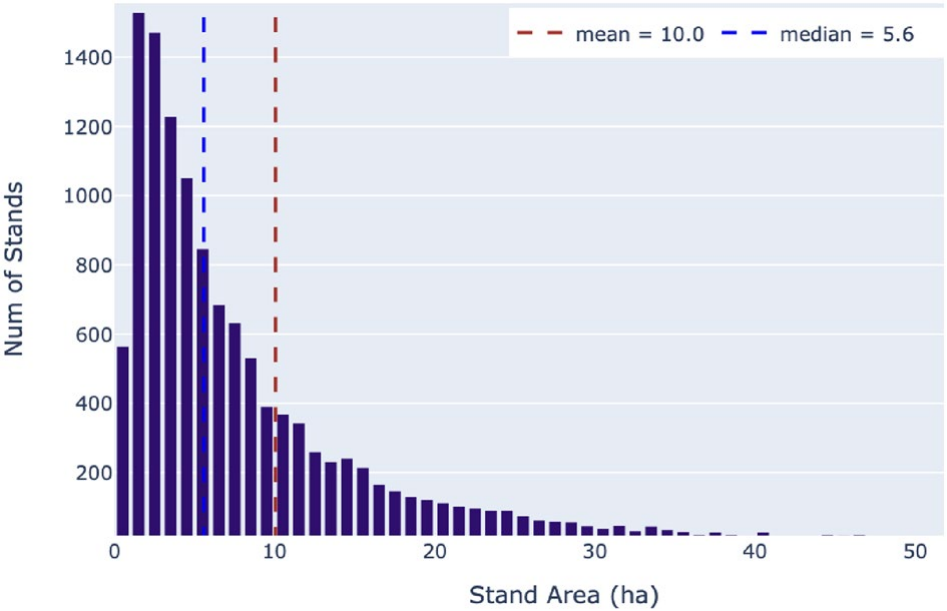
Distribution of stands by areas.

The distribution of stands by areas is shown in Fig. 3. The size of the stands varies from 0.1 to 646 ha. About 95% of the stands are smaller than 32 ha, and half of them are smaller than 5.6 ha. Since the Sentinel-2 data used has a spatial resolution of 10 m, an image patch of 10 by 10 pixels corresponds to an area of 1 hectare. Therefore, half of the stands can be described using 560 pixels or less.

**Figure 4:**
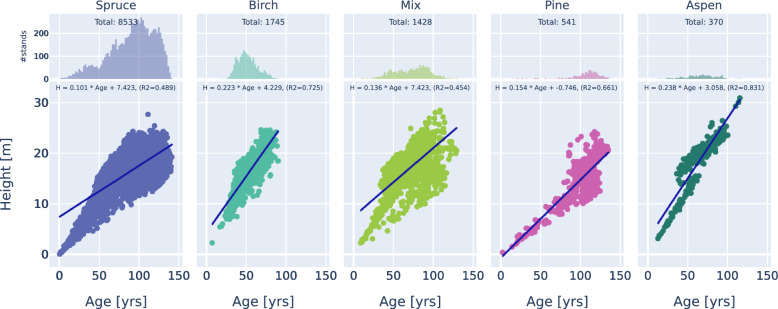
Correlation between age and height for each forest species in inventory data.

According to forest inventory data, the age distribution is heterogeneous, as can be seen in Fig. 6: there are many stands with ages over 100 years in the north, while there are very few such stands in the south.

There is a correlation between age and tree height, and the relationship is different for different tree species. The correlation between tree height and age is more pronounced with older trees, while for younger trees it is well-aligned with a straight line (Fig. 4). This correlates well with the study^22^, which notes that the relationship between height and age weakens for trees over 100 years old.

According to Fig. 4, the height and age correlation in birch, aspen, and pine stands can potentially be estimated through a linear function, albeit mainly for young stands. In spruce-dominated or mixed forests, however, the correlation is not linear. Therefore, measuring stand age by height alone is inadequate for different species and age classes.

**Figure 5:**
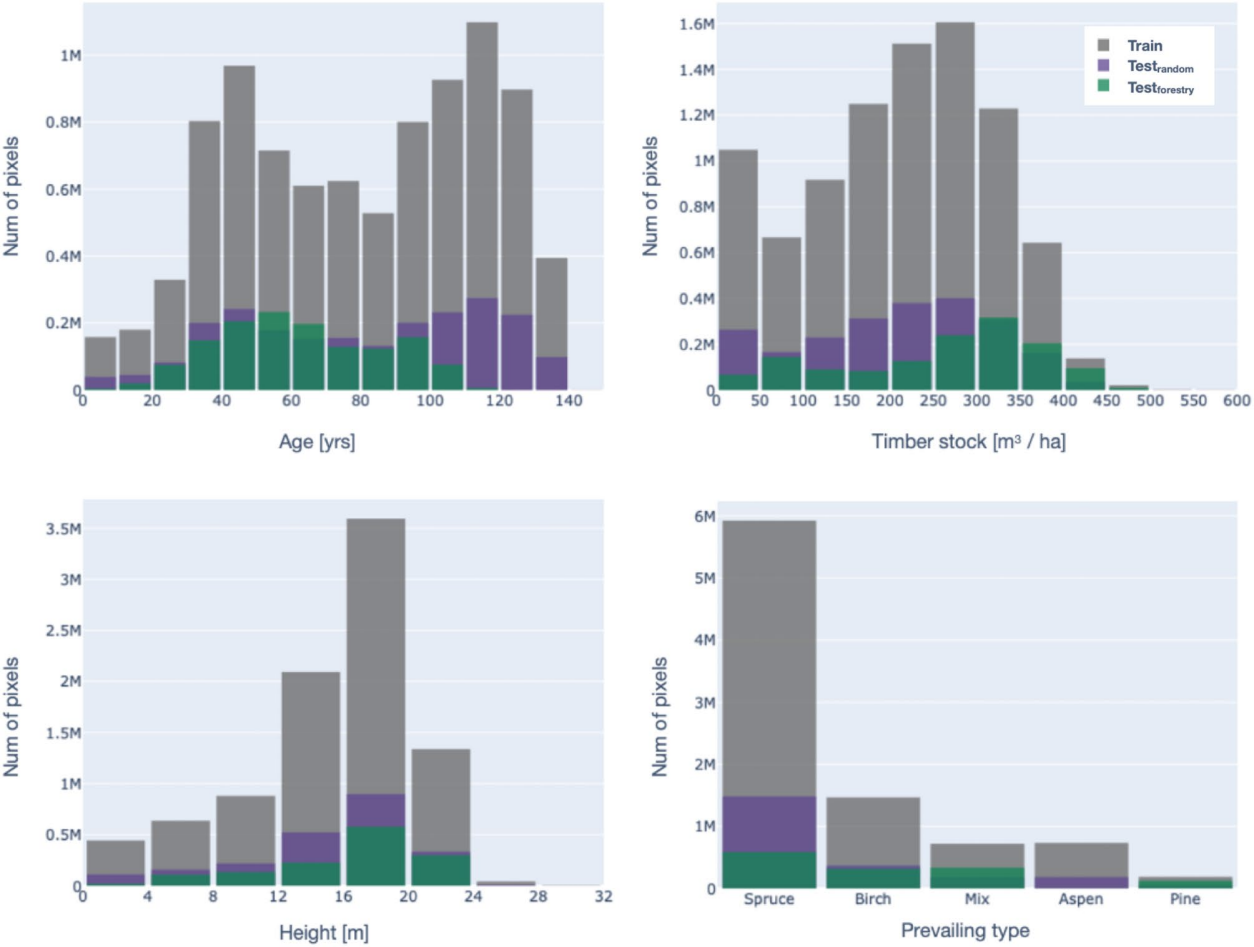
Distribution of age, dominant species, heights, and timber stock in rasterized inventory data.

To enable further analysis and the application of machine learning techniques, the vector forest inventory data is rasterized to a spatial resolution of 10 m, which matches the spatial resolution of Sentinel-2 satellite imagery. This was done with Rasterio^23^, a Python library used for reading and writing geospatial raster data. The pixel of the resulting raster inherits the value of the polygon that owns the center of the pixel. If the center of the pixel lies exactly on the boundary of the vector, then one of the equal values of the adjacent polygons is taken. In any case, there are very few edge pixels compared to the entire polygon size, and any mistakes on boundaries can be ignored. After rasterizing, we get a set of maps with pixel values equal to the corresponding value in vector format, for example, age map or dominant species map. More details about the distribution of the rasterized inventory data are depicted in Fig. 5.

### Test splits

**Figure 6:**
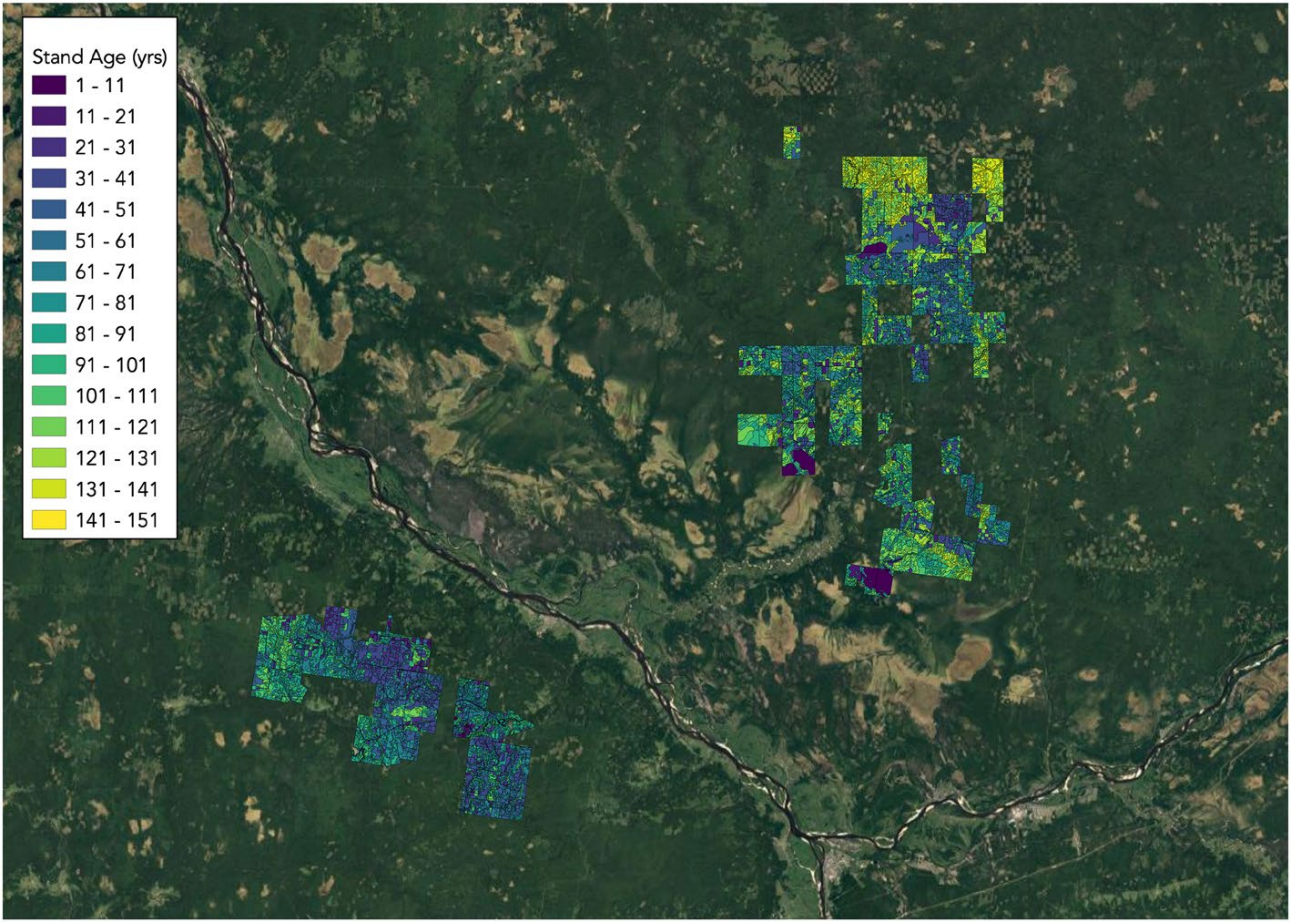
Forest age distribution from inventory data throughout the study area displays non-uniformity, with older stands being predominant in the north and younger ones in the south. The map was generated with the QGIS v.3.14 software (https://qgis.org/en/site/) and RGB satellite composite from Google Maps layers available in QGIS.

Forest characteristics are distributed non-uniformly, for example, as it is shown in Fig. 6, there are a lot of old stands on the north comparing to the south territories. This spatial heterogeneity was the reason why two test sets ($\text {Test}_{\text {forestry}}$ and $\text {Test}_{\text {random}}$) were chosen to evaluate the quality of the models. To validate the case of age prediction on new territories, we are using $\text {Test}_{\text {forestry}}$ corresponding to a separate forestry. To check model quality on the same species distribution as in train and validation sets, we use $\text {Test}_{\text {random}}$. It is collected from random pixels covering Region 1 and Region 2. Specifically, we divided pixels corresponding to Region 1 and Region 2 randomly into three parts: train (for model training), validation (for tuning hyperparameters of the model) and $\text {Test}_{\text {random}}$ in the proportion 0.68, 0.12 and 0.2, respectively. The $\text {Test}_{\text {forestry}}$ area is 13–754 hectares. Region 1 and Region 2 have a combined area of 112–887 hectares.

### Sentinel-2 data and digital elevation model

In this study, we used multispectral Sentinel-2 data with Level-2A (L2A) preprocessing that includes atmospheric correction. 10 spectral bands (B02, B03, B04, B05, B06, B07, B08, B8A, B11, B12) and SCL mask (Scene Classification Layer) with spatial resolution of 10 m were used. Images in channels B05, B06, B07, B8A, B11, B12 and SCL mask have source resolution 20 m, so they were upsampled with nearest neighbour interpolation method to 10 meters resolution via Copernicus Open Access Hub^24^. 10 bands were downloaded in digital numbers (DN) format (unitless), so that bands contain pixel values of the source data ($\text {DN} = \text {reflectance} \times 10^4$), float32 precision. SCL mask has 12 possible integer values.

For training and testing, we filtered data and used only those pixels that had value 4 in SCL mask, which corresponds to vegetation class. By this step we tried to filter non-forest pixels, specifically, cloud pixels. With a reasonable degree of accuracy, non-forest regions can be excluded through the described method, since satellite image pixels from this region correspond either to forest, deforestation areas, or clouds. There are two main reasons for this. Firstly, the area of interest is situated far away from any settlements or other non-forest land features such as crop fields. Therefore, all vegetation in this area is only forests, nothing else. Secondly, forest is only inspected during the growing season; consequently, those areas should have a leafy appearance and be in class 4 on the SCL mask. Alternatively, a more detailed forest mask can be obtained using deep learning segmentation method based on Sentinel-2 images that was in our previous study^25^.

There are two main restrictions for choosing the date of satellite image: (1) absence of cloud cover, (2) months of vegetation period (June, July, August, September). The manually selected data for the study are summarised in Table 2.

As an auxiliary data, we also used Digital Elevation Model (DEM) measurements available in Ref.^26^. The original spatial resolution is 1 m, downsampled to 10 m to match Sentinel 2 raster imagery using the rasterio.warp module (nearest neighbour resampling method).

**Table 2:** Dates of selected Sentinel-2 images for each studied region.

Date	${\text {Test}}_{{forestry}}$	Region 1	Region 2
2018-06-15			$\checkmark$
2018-07-23			$\checkmark$
2018-07-30	$\checkmark$	$\checkmark$	$\checkmark$
2018-07-31		$\checkmark$	
2018-08-04	$\checkmark$	$\checkmark$	$\checkmark$
2018-08-27	$\checkmark$	$\checkmark$	$\checkmark$
2018-09-11	$\checkmark$	$\checkmark$	$\checkmark$
2019-06-08	$\checkmark$	$\checkmark$	$\checkmark$
2019-06-13	$\checkmark$	$\checkmark$	$\checkmark$
2019-07-18			$\checkmark$
2019-08-30		$\checkmark$	
2020-06-04	$\checkmark$	$\checkmark$	$\checkmark$
2020-06-15		$\checkmark$	
2020-07-09	$\checkmark$	$\checkmark$	$\checkmark$
2020-07-20		$\checkmark$	
2020-08-21	$\checkmark$	$\checkmark$	
2021-06-02	$\checkmark$		
2021-06-07	$\checkmark$		
2021-07-04	$\checkmark$		
2021-08-28	$\checkmark$		

### Vegetation indices

Vegetation indices are known as important additional features for forest characteristics prediction^27^. In this study, we also computed a number of indices (Table 3):

- Normalized difference vegetation index (NDVI) is the standard vegetation index assessing whether or not the target being observed contains live green vegetation.
- Normalized difference water index (NDWI) measures the relative water content in vegetation and soil based on the difference in the absorption features of water and chlorophyll in the near-infrared region.
- Soil-adjusted vegetation index (SAVI) is an enhancement of NDVI for highly vegetated areas, which reduces the influence of the soil on the vegetation detection^28^. For SAVI was used the value of the constant $L = 0.428$.
- Atmospherically resistant vegetation index (ARVI) is an improvement on NDVI that minimizes its influence on atmospheric information contained in the blue channel^28^. For ARVI was used the value of the constant $y = 0.106$.
- Enhanced vegetation index (EVI) is an index that mitigates the effect of atmospheric influence on the remotely sensed signal and improves accuracy in determining vegetation coverage.

**Table 3:** Vegetation indices considered in the study.

Abbreviation	Name	Definition	Sentinel 2 formula
NDVI	Normalized difference vegetation index	$\frac{\text {NIR} - \text {RED}}{\text {NIR} + \text {RED}}$	$\frac{\text {B08} - \text {B04}}{\text {B08} + \text {B04}}$
NDWI	Normalized difference water index	$\frac{\text {NIR} - \text {SWIR}}{\text {NIR} + \text {SWIR}}$	$\frac{\text {B08} - \text {B11}}{\text {B08} + \text {B11}}$
SAVI	Soil adjusted vegetation index	$\frac{\text {NIR} - \text {RED}}{\text {NIR} + \text {RED} + L} \cdot (1.0 + L)$	$\frac{\text {B08} - \text {B04}}{\text {B08} + \text {B04} + L} \cdot (1.0 + L)$
ARVI	Atmospherically resistant vegetation index	$\frac{\text {NIR} - \text {RED} - y(\text {RED} - \text {BLUE})}{\text {NIR} + \text {RED} - y(\text {RED} - \text {BLUE})}$	$\frac{\text {B8A} - \text {B04} - y(\text {B04} - \text {B02})}{\text {B8A} + \text {B04} - y(\text {B04} - \text {B02})}$
EVI	Enhanced vegetation index	$2.5 \cdot \frac{\text {NIR} - \text {RED}}{\text {NIR} + 6 \cdot \text {RED} - 7.5 \cdot \text {BLUE}}$	$2.5 \cdot \frac{\text {B08} - \text {B04}}{\text {B08} + 6 \cdot \text {B04} - 7.5 \cdot \text {B02}}$

### Experiments

In this study, we aim at formulating and investigating a set of possible options to develop an ML-based pipeline for forest age prediciton through Sentinel-2 data. The details are described below in “Experimental” sections. In terms of ML task definition, the task is to solve a regression problem based on various spatial features.

As ML model, we chose a CatBoost algorithm available in a high-performance open-source library^29^. CatBoost is based on gradient boosting on decision trees. During training, a set of decision trees is built consecutively so that each successive tree is built with reduced loss compared to the previous trees.

Catboost is known for its speed and accuracy, it is widely used for tabular data in environmental studies^30^. It has a fast implementation on both CPU and GPU. Compared to standard Random Forest from sklearn or gradient boosting realisations such as LightGBM or XGBoost, Catboost is tens of times faster if only CPU is available, and hundreds of times faster if GPU is available^31^. This means that for a large area with many pixels, one can get results with the same or better accuracy in a foreseeable time. This in turn allows for quick experimentation with different setups and input features for forest characteristics prediction. Therefore, the Catboost algorithm optimally serves the main goal of this study as a standardised ML algorithm.

**Figure 7:**
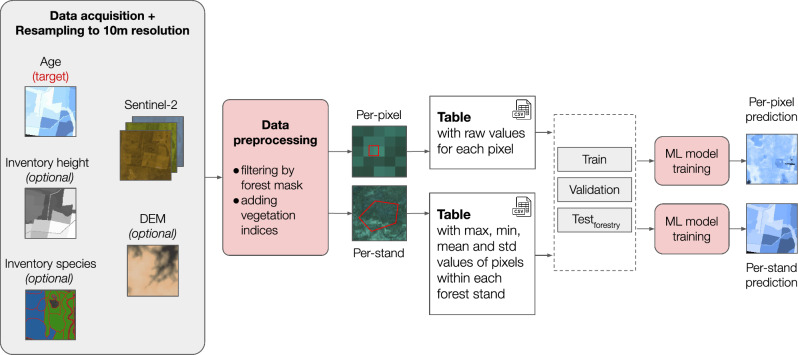
Study workflow. A set of input spatial features are considered to predict forest age. The workflow includes per-pixel and per-stand approaches to create training set based on inventory measurements.

The study workflow is depicted in Fig. 7. It involves Sentinel-2 images and a set of supplementary data. The target variable is forest age derived from field-based measurements. Per-pixel or per-stand approaches can be utilized to create a dataset. We examine both of them. Data filtering is required to exclude irrelevant samples with corrupted values. Two types of test choosing is considered and discussed. The following sections describe details of the main approaches that we studied.

#### Evaluation metrics

To evaluate prediction results for different experimental setups, we considered the following commonly used metrics: $R^2$ (Coefficient of Determination), Mean Absolute Percentage Error (MAPE), Root of Mean Squared Error (RMSE), and Mean Absolute Error (MAE). They are computed in per-pixel manner using the next formulas:

1 $$\begin{aligned}{} & {} R^2(y, \hat{y})=1-\frac{\sum _{i=1}^{n_{\text{pixels } }}\left( y_i-\hat{y}_i\right) ^2}{\sum _{i=1}^{n_{\text{pixels } }}\left( y_i-\bar{y}\right) ^2}, \end{aligned}$$

2 $$\begin{aligned}{} & {} {\text {MAPE}}(y, \hat{y})=\frac{1}{n_{\text{pixels } }} \sum _{i=1}^{n_{\text{pixels } }} \frac{\left| y_i-\hat{y}_i\right| }{\max \left( \varepsilon ,\left| y_i\right| \right) }, \end{aligned}$$

3 $$\begin{aligned}{} & {} {\text {RMSE}}(y, \hat{y})=\sqrt{\frac{1}{n_{\text{pixels } }} \sum _{i=1}^{n_{\text{pixels } }}\left( y_i-\hat{y}_i\right) ^2}, \end{aligned}$$

4 $$\begin{aligned}{} & {} {\text {MAE}}(y, \hat{y})=\frac{1}{n_{\text{pixels } }} \sum _{i=1}^{n_{\text{pixels } }}\left| y_i-\hat{y}_i\right| , \end{aligned}$$

where $\hat{y_i}$ is the predicted value of the *i*-th pixel and $y_i$ is the corresponding true value ($\varepsilon$ is an arbitrary small yet strictly positive number to avoid undefined results when *y* is zero). Using pixel-level metrics enables us to consider prediction errors weighted by the area of forest stands.

#### Experiment 1: stand-based and pixel-based approaches

In this experiment, we compare two main approaches for forest characteristics prediction using multispecral images only: stand-based and pixel-based.

In stand-based approach, we use entire stand characteristics to create a single training sample based on statistical values (min, max, std, and mean). Thus, for each forest stand, we extracted the minimum, maximum, standard deviation, and mean value of raster pixels that fall into each stand polygon. This was done for all multispectral and auxiliary data stored in raster format. In this setting, we implicitly use information about borders in the forestry, since we average multispectral and supplemented values across each stand. The obtained training sample is a forest stand. In pixel-based approach, each pixel is treated independently to the stand it belongs. The training sample in this case is a single pixel with features corresponding to Sentinel-2 and auxiliary data.

We compare three main scenarios for age prediction:

- train on stands, predict on stands, or *stand-to-stand* $\left( \blacksquare \longrightarrow \blacksquare \right)$;
- train on stands, predict on pixels, or *stand-to-pixel* $\left( \blacksquare \longrightarrow \cdot \right)$;
- train on pixels, predict on pixels, or *pixel-to-pixel* $\left( \cdot \longrightarrow \cdot \right)$.

The setup of the experiment is as follows.

##### Territories

Regions 1 and 2 are used as training and validation sets. There is no $\text {Test}_{\text {random}}$ set as it could not be properly introduced for stand based approaches, so evaluation metrics (${\text {MAPE}}$, ${\text {RMSE}}$, ${\text {MAE}}$, $R^2$) are calculated on $\text {Test}_{\text {forestry}}$ set.

##### Dates

We have carefully constructed the train and test sets to ensure that our model can generalize well to new data. Specifically, in the train set, we have excluded one date per year so that the model can learn to predict tree ages across multiple dates. Additionally, we have excluded all images from the year 2021 in the train set to evaluate how well the model can generalize to new years. To create ground truth age values, we used rasterized field inventory data observed in 2018 and added 1.0, 2.0, and 3.0 years per pixel for 2019, 2020, and 2021, respectively. This ensures that our ground truth data accurately reflects the age of each tree in the images.

##### Stand-to-stand approach $\blacksquare \longrightarrow \blacksquare$

In the stand-to-stand approach, the training tabular data was generated as follows. First, based on rasterized inventory data, pixels corresponding to each forest stand were selected. For each stand, four statistics (standard deviation, mean, minimum, and maximum values) were calculated for each individual input feature (such as spectral bands and vegetation index). The statistics for different dates were then stacked together as a single sample in the dataset.

Then, on prediction stage for given stand in test dataset, we make a prediction of stand age based on averaged statistics calculated out of pixels corresponding to the given stand. The metrics are always calculated per-pixel.

##### Stand-to-pixel approach $\blacksquare \longrightarrow \cdot$

The training set is prepared as in the previous approach; the difference is in the testing stage. In this setting, we treat each pixel independently from stand boundaries in test dataset and apply the same model trained on stand characteristics to each pixel. In this case, mean, maximum, and minimum values equal to pixel values itself, and standard deviation equals to 0.

##### Pixel-to-pixel approach $\cdot \longrightarrow \cdot$

The training set consist of pixel values for all 10 bands. In the testing stage, age was predicted in a per-pixel manner.

#### Experiment 2: aates for training

A common choice to validate an ML-based approach for forest characteristic estimation on a local scale is to use the same satellite image covering neighborhood territory. In Ref.^32^, it has been shown that such an approach can lead to inappropriate results for new observation dates even if they are close to the training date. To examine forest age prediction for various dates, we conducted an additional experiment.

The objective of this experiment is to assess the reliability of the algorithm across various dates. The experimental setup involved training a CatBoost model for each available date in a pixel-to-pixel setting, and calculating the metrics for the model on the remaining dates. This training and validation procedure was repeated for all available images.

For our experiment, we utilized multispectral imagery data. To determine the stand age values for 2019 and 2020, we used ground truth data from raster field inventory observations conducted in 2018, and added one or two years to each pixel to account for the time elapsed. This allowed us to accurately assess changes in the stands over time.

#### Experiment 3: forest species

In this experiment, our aim was to understand model capability to predict forest age on several forest species individually. We compared a model trained using age measurements for all dominant species and 5 individual models trained only on stands of each given dominant species (spruce, birch, aspen, pine, or mixed). In this experiment, we used pixel-to-pixel approach with Sentinel-2 bands and DEM for only one date (2018-07-30). It is important to recognize that in the experiment involving five regressors, the amount of training data available for each regressor was reduced. However, this reduction in data size was counterbalanced by an increase in data purity, as each regressor was trained exclusively on the pixels belonging to a particular tree specimen.

#### Experiment 4: choosing bands and indices

The objective of this experiment was to investigate the impact of additional input features on the accuracy of the prediction. We conducted a pixel-to-pixel analysis, comparing various combinations of available data. We used images from a single date (2018-08-27). Forest species information, categorized as Type of Stand, was included as a categorical feature based on inventory data.

## Results

### Experiment 1: stand-based and pixel-based approaches

In this experiment, we compared different approaches to create training samples for forest age prediction when inventory data is used as a ground-truth values. Results for stand-to-stand, stand-to-pixel, and pixel-to-pixel approaches are presented in Tables 4, 5 and 6, respectively.

**Table 4:** Experiment with the stand-to-stand approach ($\blacksquare \longrightarrow \blacksquare$ approach).

Test dates	${R^2}$	MAPE	RMSE (years)	MAE (years)
**2018-07-30**	**0.946**	**0.13**	**7.74**	**5.7**
**2018-08-04**	**0.945**	**0.125**	**7.66**	**5.6**
2018-08-27	0.814	0.421	23.25	19.4
**2019-06-08**	**0.925**	**0.148**	**9.05**	**6.5**
2019-06-13	0.920	0.155	10.09	7.9
**2020-06-04**	**0.896**	**0.167**	**11.32**	**8.4**
**2020-07-09**	**0.948**	**0.114**	**7.45**	**5.3**
2020-08-21	0.939	0.119	8.15	6.1
2021-06-02	0.907	0.145	9.91	7.4
2021-06-07	0.912	0.156	11.56	9.0
2021-07-04	0.936	0.156	11.03	8.9
2021-08-28	0.846	0.290	16.77	13.6
Average	0.91	0.18	11.2	8.7

Bold values correspond to dates that are both in train and in test sets. Only multispectral features were used for this experiment.

**Table 5:** Experiment with stand-to-pixel approach ($\blacksquare \longrightarrow \cdot$ approach).

Test dates	${R^2}$	MAPE	RMSE (years)	MAE (years)
**2018-07-30**	**0.453**	**0.285**	**17.2**	**8.2**
**2018-08-04**	**0.452**	**0.280**	**17.2**	**8.1**
2018-08-27	− 0.402	0.489	27.5	13.9
**2019-06-08**	**0.419**	**0.281**	**17.7**	**8.3**
2019-06-13	0.5	0.236	16.4	7.6
**2020-06-04**	**0.301**	**0.292**	**19.4**	**9.2**
**2020-07-09**	**0.459**	**0.274**	**17.1**	**8.3**
2020-08-21	0.423	0.258	17.6	8.2
2021-06-02	0.488	0.224	16.6	7.7
2021-06-07	0.448	0.228	17.3	8.0
2021-07-04	0.484	0.217	16.7	7.8
2021-08-28	− 0.204	0.435	25.5	12.6
Average	0.32	0.29	18.9	9.1

Bold values correspond to dates that are both in train and in test sets. Only multispectral features were used for this experiment.

**Table 6:** Experiment with pixel-to-pixel approach ($\cdot \longrightarrow \cdot$ approach).

Test dates	${R^2}$	MAPE	RMSE (years)	MAE (years)
**2018-07-30**	**0.824**	**0.224**	**14.4**	**10.9**
**2018-08-04**	**0.812**	**0.213**	**14.2**	**10.6**
2018-08-27	0.728	0.316	19.5	15.3
**2019-06-08**	**0.779**	**0.246**	**15.6**	**11.8**
2019-06-13	0.796	0.204	14.5	10.8
**2020-06-04**	**0.761**	**0.285**	**19.1**	**14.7**
**2020-07-09**	**0.830**	**0.191**	**13.4**	**9.9**
2020-08-21	0.796	0.192	14.2	10.5
2021-06-02	0.771	0.211	15.1	11.4
2021-06-07	0.797	0.192	15.2	11.3
2021-07-04	0.829	0.174	13.7	10.1
2021-08-28	0.713	0.313	20.0	15.7
Average	0.79	0.23	15.7	11.9

Bold values correspond to dates that are both in train and in test sets. Only multispectral features were used for this experiment.

The stand-to-stand approach produces the best results for both $R^2$ and MAPE, with an average $R^2$ value of 0.91 and an average MAPE of 0.18. However, this approach has a major disadvantage in that it relies on stand boundaries, which may not be available for new areas. In this regard, the stand-to-pixel and pixel-to-pixel approaches are more appropriate. The pixel-to-pixel approach yields better results in terms of both MAPE and $R^2$, with an average MAPE of 0.23 and an average $R^2$ of 0.79, compared to an average MAPE of 0.29 and an average $R^2$ of 0.32 for the stand-to-pixel approach. These findings suggest that using statistics calculated over a stand is not sufficient to predict forest age in a per-pixel manner with enough accuracy.

We should note that although there are no images from the entire year of 2021 in the training set, we can still obtain acceptable results for these dates. For instance, in a pixel-to-pixel setup, the model achieves a mean absolute percentage error (MAPE) of 0.246 and 0.192 for 6th of August, 2019 and 7th of June, 2021, respectively. However, high results are not achieved for all dates. For example, the model fails to obtain high results for the observation gathered on August 28th, 2021, with the MAPE dropping to 0.313. A possible explanation for such model behavior is that the image was obtained at the end of summer, and the spectral characteristics changed drastically. However, a rather close date of August 21st, 2020, which also did not appear in the training dataset, has a MAPE of 0.192.

Moreover, the dates with poor prediction quality are the same for all approaches. There are some dates (such as 27th of August, 2018, and 28th of August, 2021) that are not present in the training set, and for which the predictions are still poor for all three approaches. Thus, for 27th of August, 2018, stand-to-stand, stand-to-pixel, and pixel-to-pixel approaches yield just MAPE of 0.412, 0.489, and 0.316, respectively. This means that poor predictions may go unnoticed if the date of the satellite image to be predicted is not included in the training set. The effects of different choices for the training and testing dates will be explored more in the next experiment.

### Experiment 2: dates for training

**Figure 8:**
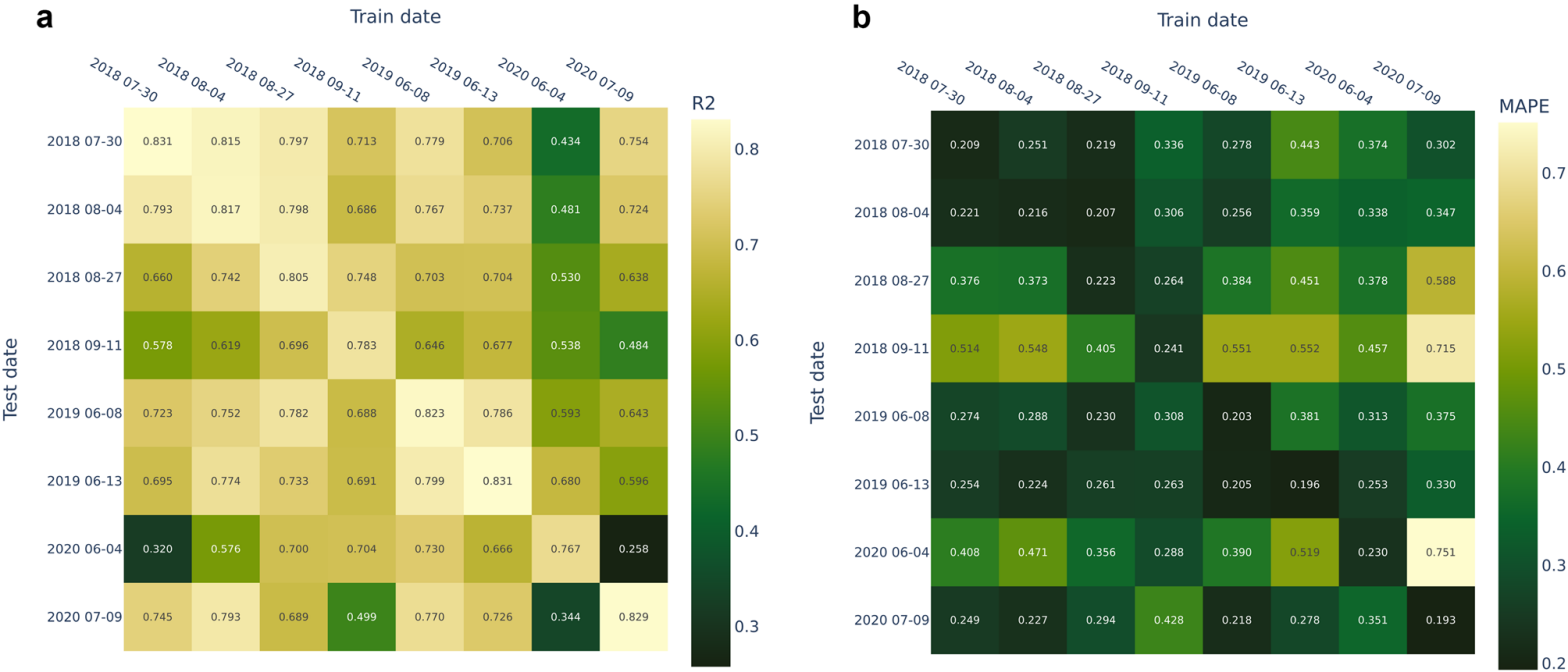
Experiment with different training and testing dates. Multispectral data were used for this experiment. (**a**) $R^2$ (the ligther the better), (**b**) mean average percentage error (the darker the better).

In this experiment, we examined different sensing dates and model capability to predict forest age for previously unseen dates. For each date, we trained an individual model and then evaluated it on all other dates. Therefore, test samples present both previously unseen regions and spectral features from new dates. We used pixel-to-pixel setup and validated the results on $\text {Test}_{\text {forestry}}$ set. Experimental results are presented in Fig. 8. The main diagonal represents testing on the same date used in training. In most cases, these values are the best. The average MAPE for the diagonal values is 0.124. However, there are a few dates (11th of September, 2018, and 4th of June, 2020) where predictions are poor for all test dates, unless the training date coincides with the test date. The average MAPE computed for the test date of 4th of June, 2020, equals to 0.257, while when we train and validate the model using this single image the MAPE equals to 0.129.

**Figure 9:**
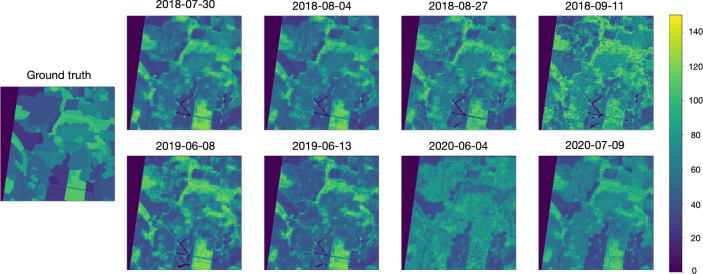
Experiment with different training and testing dates. Ground-truth and predictions for each test date in window of 300 px $\times$ 300 px (3 km $\times$ 3 km). Multispectral data were used in this experiment. The train date is 2018-07-30.

In general, the quality of the prediction is higher when the test and training dates coincide. Prediction examples for different dates are depicted in Fig. 9.

### Experiment 3: forest species

In this experiment, we aim to assess how dominant species affect predictions. We compared a single model trained employing age measurements across all prevalent species (Table 7), with five distinct individual models (Table 8) trained exclusively on stands characterized by each respective dominant species, namely spruce, birch, aspen, pine, and mixed-species stands. For these experiments, we applied the pixel-to-pixel setup.

The overall MAPE for a single regressor is 0.197, while for separate regressors we have an average MAPE of 0.126 and a maximum MAPE of 0.184. Thus, having a separate regressor for each tree specimen reduces the MAPE.

We can observe that learning patterns from a dataset of all species is more challenging for the model compared to a dataset of a single specimen. For instance, age prediction for birch and spruce is more accurate when predictions are made by an individual model rather than a shared model for all species. This trend is observed for both the $\text {Test}_{\text {random}}$ and $\text {Test}_{\text {forestry}}$ sets. Additionally, a specific model for pine and aspen performs better in $\text {Test}_{\text {random}}$, but not in $\text {Test}_{\text {forestry}}$ due to the insufficient amount of stands with these dominant species in the $\text {Test}_{\text {forestry}}$ set.

However, this does not apply to Mix stands. For this complex type of stands, it is irrelevant whether the train set has become homogeneous or not. In fact, the predictions for this type of stand became worse because the train set decreased.

**Table 7:** Experiment for different forest species.

(a) ${\text {Test}}_{{\text {random}}}$						(b) ${\text{Test}}_{\text{forestry}}$					
${\text {Test}}_{{\text {random}}}$	${R^2}$	MAPE	RMSE (years)	MAE (years)	Support (#pixels)	${\text{Test}}_{\text{forestry}}$	${R^2}$	MAPE	RMSE (years)	MAE (years)	Support (#pixels)
Spruce (1)	0.915	0.207	14.8	10.5	1,072,973	Spruce (1)	0.844	0.246	15.4	11.6	590,042
Birch (2)	0.680	0.180	12.2	8.6	2,624,220	Birch (2)	0.515	0.217	13.8	10.3	318,679
Mix (3)	0.814	0.167	14.2	10.7	130,920	Mix (3)	0.755	0.222	14.7	11.3	334,346
Pine (4)	0.859	0.186	17.4	12.1	133,460	Pine (4)	− 0.554	0.218	23.0	20.3	3444
Aspen (5)	0.796	0.181	12.4	9.5	34,623	Aspen (5)	0.694	0.204	15.2	11.9	124,304
Overall	0.911	0.197	14.5	10.3	1,634,398	Overall	0.805	0.229	14.9	11.3	1,370,815

One model for all species. Multispectral and DEM data were used.

**Table 8:** Experiment for different forest species.

(a) ${\text {Test}}_{{\text {random}}}$						(b) ${\text{Test}}_{\text{forestry}}$					
${\text {Test}}_{{\text {random}}}$	${R^2}$	MAPE	RMSE (years)	MAE (years)	Support (#pixels)	${\text{Test}}_{\text{forestry}}$	${R^2}$	MAPE	RMSE (years)	MAE (years)	Support (#pixels)
Spruce (1)	0.922	0.184	14.2	10.0	1,073,297	Spruce (1)	0.857	0.242	15.2	11.2	590,042
Birch (2)	0.814	0.110	7.1	5.4	262,493	Birch (2)	0.568	0.133	8.1	6.3	318,679
Mix (3)	0.893	0.122	10.5	7.8	131,004	Mix (3)	0.721	0.235	15.0	11.6	334,346
Pine (4)	0.933	0.127	11.8	7.9	132,750	Pine (4)	− 0.232	0.205	21.0	18.4	3444
Aspen (5)	0.942	0.087	6.7	4.5	34,856	Aspen (5)	0.512	0.256	19.6	14.5	124,304
Average	0.90	0.13	10.1	7.1	–	Average	0.48	0.214	15.8	12.4	–

An individual model is trained for each specimen. Multispectral and DEM data were used.

### Experiment 4: choosing bands and indices

This experiment compares different feature sets to understand which source of information is more significant for predicting the age of a forest. We trained an ML model using different subsets of features and compared the results on both the $\text {Test}_{\text {random}}$ and $\text {Test}_{\text {forestry}}$ sets. Tables 9 and  10 show the corresponding results.

**Table 9:** Experiment with different input features.

Features	${R^2}$	MAPE	RMSE (years)	MAE (years)
$\text{Height}_{\text{stand}}$	0.761	0.254	22.8	18.3
$\text{Height}_{\text{stand}} + \text{B08}$	0.885	0.165	16.4	12.0
$\text{Height}_{\text{stand}} + \text{B03}$	0.793	0.231	21.4	16.8
*B*02...*B*12	0.893	0.223	15.8	11.5
$\text{B02}...\text{B12} +$ vegetation indices	0.896	0.221	15.6	11.3
$\text{B02}...\text{B12} + \text{DEM}$	0.897	0.219	15.6	11.2
$\text{Height}_{\text{stand}} + \text{B02}...\text{B12}$	0.951	0.104	10.8	7.6
$\text{Height}_{\text{stand}} + \text{B02}...\text{B12} + \text{DEM}$	0.956	0.101	10.4	7.2
${\textbf {Height}}_{{\textbf {stand}}} + {\textbf {Type}}_{{\textbf {stand}}} + {\textbf {B02}}...{\textbf {B12}} + {\textbf {DEM}}$	$\mathbf {0.986}$	$\mathbf {0.080}$	**8.8**	**6.0**

The results are declared for ${Test}_{{random}}$.

The highest model results are in bold.

**Table 10:** Experiment with different input features.

Features	${R^2}$	MAPE	RMSE (years)	MAE (years)
$\text{Height}_{\text{stand}}$	0.739	0.416	27.5	22.6
$\text{Height}_{\text{stand}} + \text{B08}$	0.831	0.255	18.5	14.1
$\text{Height}_{\text{stand}} + \text{B03}$	0.753	0.371	24.9	20.1
*B*02...*B*12	0.805	0.223	14.6	11.0
$\text{B02}...\text{B12} +$ vegetation indices	0.834	0.211	13.9	10.5
$\text{B02}...\text{B12} + \text{DEM}$	0.801	0.233	14.9	11.3
$\text{Height}_{\text{stand}} + \text{B02}...\text{B12}$	0.913	0.148	11.5	8.4
$\text{Height}_{\text{stand}} + \text{B02}...\text{B12} + \text{DEM}$	0.904	0.159	12.3	9.1
${\textbf {Height}}_{{\textbf {stand}}} + {\textbf {Type}}_{{\textbf {stand}}} + {\textbf {B02}}...{\textbf {B12}} + {\textbf {DEM}}$	$\mathbf {0.943}$	$\mathbf {0.124}$	**9.9**	**7.3**

The results are declared for ${Test}_{{forestry}}$.

The highest model results are in bold.

Knowledge of stand dominant species and stand height greatly improves the results, yielding the MAPE of 0.08 and 0.124 for $\text {Test}_{\text {random}}$ and $\text {Test}_{\text {forestry}}$. However, using multispectral data alone still provide good quality predictions, with MAPE of 0.223 for both test sets.

One of the most important features for forest age prediction is B08 band, which corresponds to the NIR spectral range with an initial resolution of 10 m. Other bands have a smaller effect on the result and for the sake of compactness we have only shown band B03 in the table. The MAPE is slightly reduced by band B03 by 0.02 for $\text {Test}_{\text {random}}$ and 0.04 for $\text {Test}_{\text {forestry}}$ (all other bands have a similar effect).

Adding vegetation indices listed in Table 3 to the Sentinel-2 data slightly reduces MAPE by 0.01 for $\text {Test}_{\text {forestry}}$ and has almost no effect for $\text {Test}_{\text {random}}$. The incorporation of NDVI, NDWI, SAVI, ARVI, and EVI yields a minor yet a positive effect on the estimation of forest age.

When combined with Sentinel-2 bands, forest height derived from inventory data ($\text {Height}_{\text {stand}}$) produces a MAPE of 0.104 and a $R^2$ of 0.951 on the $\text {Test}_{\text {random}}$ set, and a MAPE of 0.148 and $R^2$ of 0.913 on the $\text {Test}_{\text {forestry}}$ set, i.e. utilizing information about height significantly improves the age predictions.

**Figure 10:**
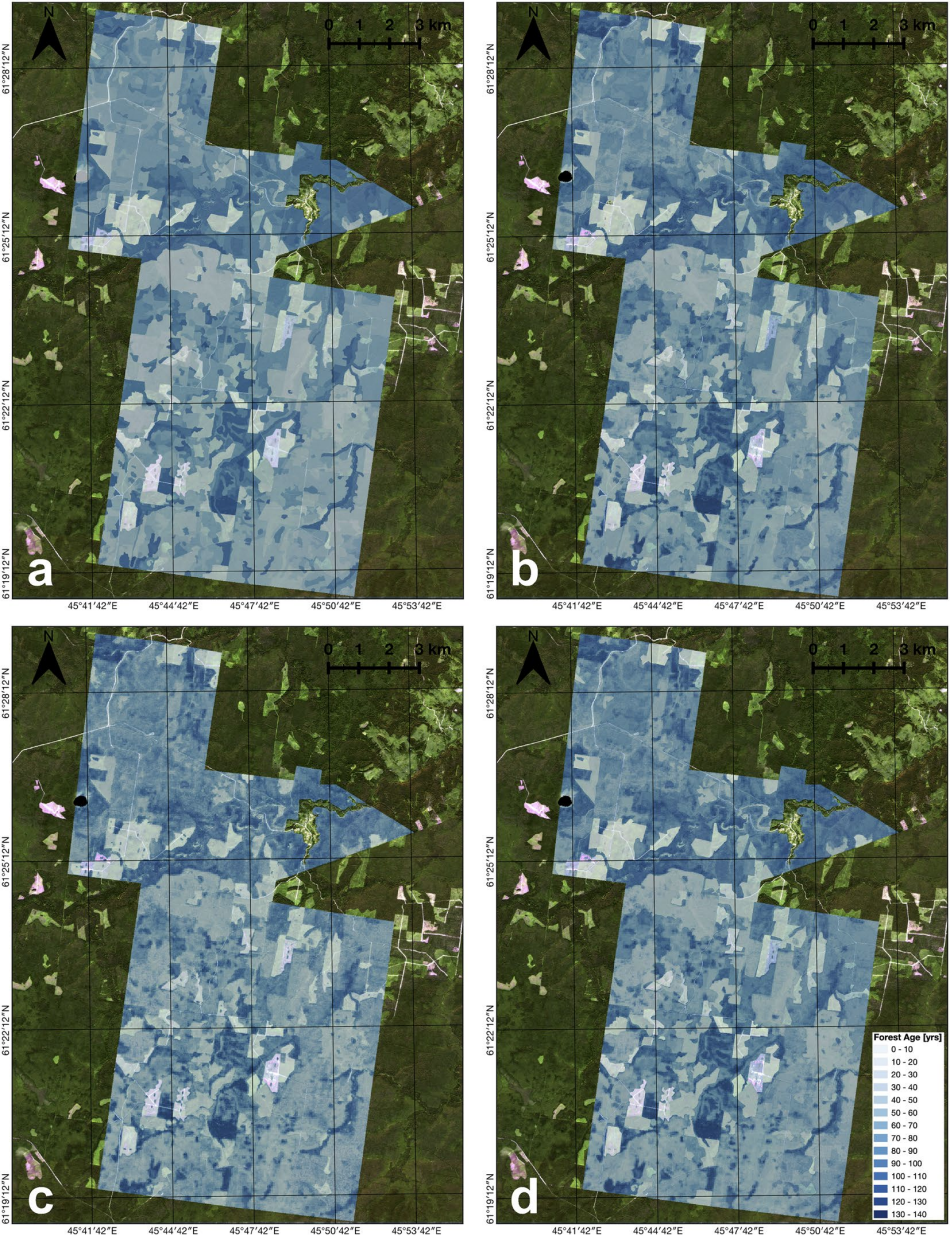
Experiment with different input features on the $\text {Test}_{\text {forestry}}$: (**a**) Ground-truth forest age; (**b**) Predictions using all the input features $\left( \text{Height}_{\text{stand}} + \text{Type}_{\text{stand}} + \text{B02}...\text{B12} + \text{DEM} \right)$; (**c**) Predictions using only multispectral images $\left( \text{B02}...\text{B12} \right)$; (**d**) Predictions using multispectral images and height from inventory data $\left( \text{Height}_{\text{stand}} + \text{B02}...\text{B12} \right)$. The background is Sentinel-2 L2A RGB image for 2018-07-30. The maps were generated with the QGIS v.3.14 software (https://qgis.org/en/site/).

Visualised ground-truth and model predictions using different input features are shown in Fig. 10. All available information gives predictions that are closest to the ground-truth. However, the use of satellite imagery alone, without any information on forest inventory properties, still gives good results for forest age prediction for the new areas.

## Discussion

### Experiment 1: stand-based and pixel-based approaches

Stand-based and pixel-based approaches are commonly used strategies to predict forestry variables. However, typically only one approach is utilized in a given study. To address this limitation, we conducted a comparative analysis of stand-based and pixel-based approaches, highlighting the respective strengths and weaknesses of each.

The per-stand approach has a main disadvantage, which is the requirement of stand boundary information that may not be available in practice. Experimental results have shown poor performance for model prediction in the stand-to-pixel setup. Nonetheless, this approach holds promise for future investigation, as it has the potential to create a robust transfer between stand-based training procedures and pixel-based inference.

In this study, we focus specifically on different ML-based pipelines for forest age estimation. A more detailed look at deep learning models applications can be inspired by the obtained findings. For instance, advanced computational techniques can be applied to conduct future comprehensive analysis with neural networks.

Previous studies have shown high performance for forest age estimation on a large scale with low spatial resolution of 1 km^12^. It is important to notice, that the present experiments are conducted with the spatial resolution of 10 m per pixel. It provides a rather detailed forest age map that can be further applied for environmental studies such as carbon stock estimation and ecological condition assessment.

### Experiment 2: dates for training

In the previous studies, it has been shown that geo-spatial distribution plays a vital role in environmental tasks^33^. When a model is designed to process new territories, it assumes that new observation dates are considered. Therefore, we studied the effect of transferring between different dates. The quality of the prediction is usually higher when for training and testing the same date is used. Nevertheless, for regions that are remote from the studied area, images with the same date of observation can be not available. We highlighted its importance for practical applications development and comprehensive study conduction. The main challenge is to identify “appropriate” images for new regions even if there is not any ground-truth measurements. The future investigation on this topic is required.

### Experiment 3: forest species

In Ref.^15^, quality of forest age estimation was measured for a single species stands, namely for spruce stands. In our study, we aimed to assess how forest species affect prediction results. The achieved results show that separate models perform better than a single shared model that predicts forest age for all forest species simultaneously. It is more difficult for the model to learn patterns from a dataset of all species than from a dataset of a single specimen. Indeed, age prediction for birch and spruce is better when predicted by a specific model rather than by a single model for all species, which could be seen in both $\text {Test}_{\text {random}}$ and $\text {Test}_{\text {forestry}}$. In summary, when a forest species map is available, it is better to use a single model for each of the given species.

### Experiment 4: choosing bands and indices

Experimental results demonstrated that knowledge of stand dominant species and stand height greatly improves the results. However, in practice, this data is not available in most cases and all forest variables should be estimated based on remote sensing observations. Therefore, the main attention should be pay to remote sensing approaches. The obtained results show high perspective to develop efficient pipelines for forest age estimation. Moreover, it encourages further development of algorithms for precise forest characteristics estimation such as height or biomass^34^. They can be based on satellite data of different properties and further integrated into an entire pipeline for forestry analysis.

Incorporating observations for different dates and creating time series might be effective for additional spectral features extraction. For instance, the achieved findings on forest age estimation can be aggregated with results for Sentinel-2 time series exploration in land use classification^35^. In addition to summer images, one can utilise winter observations for conifers as an additional source of spectral characteristics.

In summary, it is worth noting that the experimental results obtained are consistent with previous studies. However, due to limitations in open-access forest inventory datasets, it is difficult to compute metrics on the same test sets for the same regions. Directly comparing metrics is also not very informative due to differences in environmental conditions among regions. Nevertheless, the achieved results are consistent with those reported in other studies. For example, in Ref.^15^, the authors reported an RMSE of 11.5 years for spruce stands using Sentinel-2 data.

To boost model performance, additional vegetation indices can be further considered such as an adaptive vegetation index or others proposed in Refs.^36,37^. In the present experiments, we also showed the importance of NIR spectral band. A future study might consider utilizing of artificially generated NIR band^38^.

## Conclusions

The present work addresses the challenge of estimating forest age through remote sensing data. Accurate forest age maps are crucial both for environmental studies and for making informed management decisions. Developing these maps requires optimal training strategies for remote sensing observations and machine learning (ML) algorithms. The specific definition of training samples and the train-test split strategy can affect the ultimate results. Therefore, we formalized and considered a set of possible options to create an ML-based pipeline for forest age estimation based on Sentinel-2 images. We compared auxiliary features such as DEM and forest inventory information; we studied the model’s performance with two train-test split strategies and examined per-pixel and per-stand dataset sampling. Additionally, we studied the train-test image selection with different observation dates. The obtained mean absolute error (MAE) metric for per-pixel forest age estimation was 11 and 7 years for just multispectral images and for Sentinel-2 with DEM and forest inventory information, respectively.

## Data Availability

The datasets used and analysed during the current study available from the corresponding author on reasonable request.

## References

[1] Jayathunga S, Owari T, Tsuyuki S (2019). Digital aerial photogrammetry for uneven-aged forest management: Assessing the potential to reconstruct canopy structure and estimate living biomass. Remote Sens..

[2] Zema DA, Plaza-Alvarez PA, Xu X, Carra BG, Lucas-Borja ME (2021). Influence of forest stand age on soil water repellency and hydraulic conductivity in the mediterranean environment. Sci. Total Environ..

[3] Keenan RJ, Weston CJ, Volkova L (2021). Potential for forest thinning to reduce risk and increase resilience to wildfire in Australian temperate eucalyptus forests. Curr. Opin. Environ. Sci. Health.

[4] Zhao Q, Yu S, Zhao F, Tian L, Zhao Z (2019). Comparison of machine learning algorithms for forest parameter estimations and application for forest quality assessments. For. Ecol. Manag..

[5] Ivliev N (2022). First earth-imaging cubesat with harmonic diffractive lens. Remote Sens..

[6] Illarionova S (2022). A survey of computer vision techniques for forest characterization and carbon monitoring tasks. Remote Sens..

[7] Pödör, Z., Manninger, M. & Jereb, L. Application of sigmoid models for growth investigations of forest trees. In *Advanced Computational Methods for Knowledge Engineering: Proceedings of the 2nd International Conference on Computer Science, Applied Mathematics and Applications (ICCSAMA 2014)* 353–364 (Springer, 2014).

[8] Zhang C (2014). Mapping forest stand age in china using remotely sensed forest height and observation data. J. Geophys. Res. Biogeosci..

[9] Yang X, Liu Y, Wu Z, Yu Y, Li F, Fan W (2020). Forest age mapping based on multiple-resource remote sensing data. Environ. Monit. Assess..

[10] Yu Z, Zhao H, Liu S, Zhou G, Fang J, Yu G, Tang X, Wang W, Yan J, Wang G, Ma K (2020). Mapping forest type and age in China’s plantations. Sci. Total Environ..

[11] Chemura A, van Duren I, van Leeuwen LM (2015). Determination of the age of oil palm from crown projection area detected from worldview-2 multispectral remote sensing data: The case of Ejisu-Juaben district, Ghana. ISPRS J. Photogramm. Remote Sens..

[12] Zhang C (2014). Mapping forest stand age in China using remotely sensed forest height and observation data. J. Geophys. Res. Biogeosci..

[13] Wallerman, J. *et al.* Estimating forest age and site productivity using time series of 3d remote sensing data. In *2015 IEEE International Geoscience and Remote Sensing Symposium (IGARSS)* 3321–3324. 10.1109/IGARSS.2015.7326529 (2015).

[14] Zhang Y, Yao Y, Wang X, Liu Y, Piao S (2017). Mapping spatial distribution of forest age in China. Earth Space Sci..

[15] Schumacher J, Hauglin M, Astrup R, Breidenbach J (2020). Mapping forest age using national forest inventory, airborne laser scanning, and sentinel-2 data. For. Ecosyst..

[16] Frate L (2016). Spatially explicit estimation of forest age by integrating remotely sensed data and inverse yield modeling techniques. IForest.

[17] Zhang Y, Yao Y, Wang X, Liu Y, Piao S (2017). Mapping spatial distribution of forest age in China. Earth Space Sci..

[18] Reese H (2003). Countrywide estimates of forest variables using satellite data and field data from the national forest inventory. AMBIO J. Hum. Environ..

[19] Morin D (2019). Estimation and mapping of forest structure parameters from open access satellite images: Development of a generic method with a study case on coniferous plantation. Remote Sens..

[20] Spracklen B, Spracklen DV (2021). Synergistic use of sentinel-1 and sentinel-2 to map natural forest and acacia plantation and stand ages in north-central Vietnam. Remote Sens..

[21] Illarionova S, Trekin A, Ignatiev V, Oseledets I (2020). Neural-based hierarchical approach for detailed dominant forest species classification by multispectral satellite imagery. IEEE J. Select. Top. Appl. Earth Observ. Remote Sens..

[22] Maltamo M, Kinnunen H, Kangas A, Korhonen L (2020). Predicting stand age in managed forests using national forest inventory field data and airborne laser scanning. For. Ecosyst..

[23] Gillies, S. *et al.**Rasterio: Geospatial Raster i/o for Python Programmers* (2013).

[24] Copernicus Open Access Hub. https://scihub.copernicus.eu/ (Accessed 2023).

[25] Mirpulatov I, Illarionova S, Shadrin D, Burnaev E (2023). Pseudo-labeling approach for land cover classification through remote sensing observations with noisy labels. IEEE Access.

[26] ArcticDem. https://www.pgc.umn.edu/data/arcticdem/ (Accessed 2023).

[27] Zheng G, Chen J, Tian Q, Ju W, Xia X (2007). Combining remote sensing imagery and forest age inventory for biomass mapping. J. Environ. Manag..

[28] Jinguo Y, Wei W (2004). Identification of forest vegetation using vegetation indices. Chin. J. Popul. Resour. Environ..

[29] Dorogush, A. V., Ershov, V. & Gulin, A. Catboost: Gradient boosting with categorical features support. Preprint at http://arXiv.org/1810.11363 (2018).

[30] Li S (2021). Quantification of chlorophyll-a in typical lakes across china using sentinel-2 msi imagery with machine learning algorithm. Sci. Total Environ..

[31] *CatBoost—State-of-the-Art Open-Source Gradient Boosting Library with Categorical Features Support—catboost.ai*. https://catboost.ai/#benchmark (Accessed 28 October 2023).

[32] Illarionova S (2021). Mixchannel: Advanced augmentation for multispectral satellite images. Remote Sens..

[33] Illarionova S (2022). Augmentation-based methodology for enhancement of trees map detalization on a large scale. Remote Sens..

[34] Labrière N (2023). Toward a forest biomass reference measurement system for remote sensing applications. Glob. Change Biol..

[35] Campos-Taberner M (2020). Understanding deep learning in land use classification based on sentinel-2 time series. Sci. Rep..

[36] Firsov N (2021). Neural network-aided classification of hyperspectral vegetation images with a training sample generated using an adaptive vegetation index. Comput. Opt..

[37] Giovos R, Tassopoulos D, Kalivas D, Lougkos N, Priovolou A (2021). Remote sensing vegetation indices in viticulture: A critical review. Agriculture.

[38] Illarionova S, Shadrin D, Trekin A, Ignatiev V, Oseledets I (2021). Generation of the nir spectral band for satellite images with convolutional neural networks. Sensors.

